# Maternal Gestational Diabetes Is Associated with High Risk of Childhood Overweight and Obesity: A Cross-Sectional Study in Pre-School Children Aged 2–5 Years

**DOI:** 10.3390/medicina59030455

**Published:** 2023-02-24

**Authors:** Maria Mantzorou, Dimitrios Papandreou, Eleni Pavlidou, Sousana K. Papadopoulou, Maria Tolia, Maria Mentzelou, Antigoni Poutsidi, Georgios Antasouras, Georgios K. Vasios, Constantinos Giaginis

**Affiliations:** 1 Department of Food Science and Nutrition, University of the Aegean, 81400 Myrina, Greece; 2 Department of Health Sciences, College of Natural and Health Sciences, Zayed University, Khalifa B City, Abu Dhabi 144534, United Arab Emirates; 3 Department of Nutritional Sciences and Dietetics, School of Health Sciences, International Hellenic University, 57001 Thessaloniki, Greece; 4 School of Medicine, University of Crete, 71110 Heraklion, Greece; 5 Department of Surgery, Medical School, University of Thessaly, 41100 Larissa, Greece

**Keywords:** gestational diabetes, childhood obesity, preschool children, pregnancy, perinatal factors, pre-pregnancy BMI

## Abstract

*Background and Objectives*: Childhood obesity is a global public health concern with long-term and serious health implications. An important factor for childhood obesity is maternal gestational diabetes mellitus (GDM), which in turn impacts maternal and offspring long-term health. This study aimed to investigate the associations between maternal GDM and childhood weight status and multiple anthropometric and sociodemographic factors and perinatal outcomes. *Materials and Methods*: A total of 5348 children aged 2–5 years old and their paired mothers took part in the study. Questionnaires were utilized to evaluate the sociodemographic factors and perinatal outcomes as well as smoking habits, educational level, economic status, age, and parity status. Children’s anthropometric parameters were measured, and maternal medical history, preterm birth records, and anthropometric measures during pregnancy were retrieved by their medical records. *Results*: Overall, 16.4% of the children aged at 2–5 years were overweight, and 8.2% of them were affected by obesity, leading to a total 24.6% of children with overweight/obesity. Further, 5.5% of the enrolled mothers were diagnosed with gestational diabetes mellitus. GDM doubles the probability of childhood overweight/obesity at ages 2–5 years old independently of multiple confounding factors. Pre-pregnancy overweight and obesity, older maternal age, and smoking are risk factors for GDM, while GDM additionally increases the risk of preterm birth. Children of mothers that developed GDM were at greater risk of overweight or obesity, with the association between GDM and offspring’s weight status being independent of confounding factors. *Conclusions*: GDM is a severe public health issue with prolonged complications for both the mother and their children. Public health approaches and programs need to promote the negative role of pre-pregnancy weight and smoking status as well as the significance of a good glycemic control throughout gestation in women of childbearing age.

## 1. Introduction

Childhood obesity constitutes a severe public health issue worldwide, with long-term and serious health complications. The risk of childhood obesity begins in utero [1], influenced by genetic, epigenetic, and environmental factors [2,3,4]. Childhood overweight and obesity levels in European countries are high [5], while the data in pre-school stages of life remain scarce [6]. In pre-school children up to 5 years old, the incidence of overweight and obesity ranges between 1% and 28.6% [7]. In Greece, the prevalence of risk of overweight and obesity in pre-school children up to 5 years old has been estimated at 21.3% based on the International Obesity Task Force (IOTF) method of estimation [8]. Due to the physical and mental health implications of obesity as well as its economic burden, timely preventive and management strategies are strongly needed. An important factor for childhood obesity appears to be maternal gestational diabetes mellitus (GDM).

GDM affects about one in six pregnant women in the world [9] and impacts both maternal and offspring health outcomes in the short and long term. In the short term, GDM can lead to hypertension and pre-eclampsia [9] and dystocia [10,11]. In the long term, GDM enhances the likelihood of cardiometabolic, liver, and kidney diseases [9]. Women with GDM show elevated probability to be diagnosed with insulin-independent diabetes at the next stage of their life [9,11], especially those who needed insulin, had high BMI, had multiparous pregnancy, had offspring with macrosomia, and those who gained weight between their pregnancies [12]. A new meta-analysis demonstrated that advanced maternal age, family history of diabetes, Black and non-Hispanic White ethnicities, and living in Europe and Southeast Asia are associated with increased probability of diabetes after being diagnosed with GDM [13]. In fact, almost half of mothers with a history of GDM will exhibit type II diabetes at the next decade of their life [14].

Among the most important risk factors, weight status before gestation and gestational weight gain (GWG) are strongly associated with several perinatal complications for both the mothers and their fetuses, including GDM [15,16,17]. Notably, meta-analysis studies supported evidence for the presence of a dose–response association between BMI before gestation and GDM, highlighting the need for robust public health interferences for the management of maternal BMI before gestation as well as for the control of the maternal body weight gain throughout gestation [16,17]. Moreover, GDM has considerably been related with elevated childbirth weight and a high probability of large for gestational age and macrosomia [18].

Regarding fetal health, maternal GDM can lead to increased fetal adiposity [19] and macrosomia [20], which can increase the risk of difficulties and trauma during birth as well as preterm birth [10,20]. Furthermore, due to the change on glucose supply and offspring’s hyperinsulinemia, neonatal hypoglycemia may be present [21]. Apart from short-term complications, maternal GDM may cause prolonged health implications for their children, including metabolic syndrome and type II diabetes, obesity and abdominal obesity, hypertension and dyslipidemia [9,22,23,24,25], as well as cardiovascular diseases [26]. Notably, the impact of GDM on children’s glucose and insulin resistance is not solely dependent on either mother or child’s BMI status or familial history of diabetes mellitus [25], as epigenetic modifications in utero can influence long-term health outcomes [27].

However, there are not enough data concerning the potential effect of GDM on the probability of exhibiting childhood overweight and obesity in pre-school age and especially in the Greek population. In view of the above considerations, the purpose of the present cross-sectional study was to explore whether maternal GDM may affect childhood weight status at the first stages of life while taking into account multiple anthropometric and socio-demographic factors and perinatal outcomes as confounders.

## 2. Materials and Methods

### 2.1. Subjects

In the current survey, 7122 Caucasian mothers and their matched children at the age of 2–5 years old were initially, voluntarily enrolled from nine diverse Greek areas, namely Athens, Thessaloniki, Larisa, Patra, Alexandroupolis, Kalamata, Ioannina, Crete, and the North Aegean from community and especially from nursery schools and playgrounds. Enrollment to the survey took place from May 2016 to September 2020. Beyond the enrolled 7122 Caucasian mothers, another 87 mothers were selected to participate to the study; however, they refused to take part. In multiparous Caucasian mothers, merely the most recent gestation was considered for analysis. The participating mothers and their children had no disease during the postpartum period.

All recorded data were confidential. The participating mothers were informed about the objectives of the survey and signed a permission form in which they approved that their personal data may be published. Sample size estimation was established using the PS: Power and Sample Size calculator program based on achieving adequate power for hypothesis testing.

**Figure 1:**
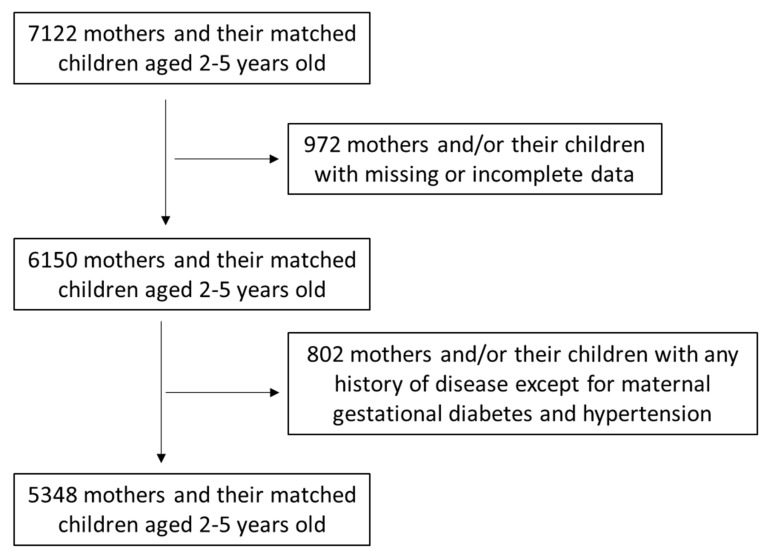
Flow chart of study enrolment.

Among the 7122 firstly enrolled women and paired children, 972 of them (13.6%) were excluded from the survey because of lost or inadequate data. Among the remaining 6150 women and paired children, 802 (13.0%) of them were omitted from the survey because of a history of disease such as diabetes mellitus type 1 or 2, hyperlipidemia, hypertension, anemia, thyroid disorders, cardiovascular diseases, osteoporosis, multiple sclerosis, polycystic ovary syndrome, inflammatory gastrointestinal diseases, gallstones, autoimmune liver disease, celiac disease, pelvic floor dysfunction, and cancer, leading to a final response rate of 75.1%. History of disease was self-reported by the participating women via the provided questionnaires. The only inclusion criterion regarding disease was gestational diabetes or pregnancy-induced hypertension that had been treated effectively. A total of 5348 women and their paired children were finally included. A flow chart of study enrolment is presented in Figure 1.

The survey was certified by the Ethical Board of the University of the Aegean (ethical authorization protocol: No. 12/14.5.2016) and was in agreement with the World Health Organization (52nd WMA General Assembly, Edinburgh, Scotland, 2000).

### 2.2. Study Design

At the time of study, 2–5 years postpartum, certified semi-quantitative questionnaires were utilized to evaluate the sociodemographic parameters and perinatal outcomes of the survey population [28,29]. Children’s anthropometric characteristics (weight and height) at the age of 2–5 years old (2–5 years postpartum) were determined by qualified nutritionists as per protocol [28,29,30,31]. Children’s weight was determined utilizing the same electronic scale, and height was determined utilizing a portable stadiometer. IOTF method was applied to classify overweight and obesity in participating children [32]. In fact, children with BMI between 85th and 95th percentiles of IOFT growth curves were categorized as overweight, and those with BMI ≥ 95th percentile of IOFT growth curves were categorized as obese [32].

Mothers’ GDM status was retrieved by their medical records. All mothers were screened for GDM by applying a universal oral glucose tolerance test (OGTT) during pregnancy [33]. In fact, a fasting OGTT after 75 g glucose with a cut-off plasma glucose of ≥140 mg/dL after 2 h for the first and subsequent trimester at 24–28 weeks of pregnancy was performed for all participating mothers [33]. Mothers’ weight and height was derived from their records with measurements during the first weeks of gestation and immediately prior to childbirth during their visit to their personal gynecologists and/or the health care units that they visited. Mothers’ pre-pregnancy body mass index (BMI) was determined according to the weight and height that was stated in their medical records. Gestational weight gain (GWG) of participating mothers was also retrieved by their medical files. In addition, participating women were questioned regarding whether they followed breastfeeding practices at all and whether they followed exclusive breastfeeding for a minimum period of four months. To avoid recall bias, the women were questioned regarding whether they exclusively breastfed for a minimum period of four months since at this moment they were counselled to slowly incorporate pulp foods to the feeding practices of their children, and thus, they memorized more accurately this time point, making their responses more consistent. On the contrary, women who followed breastfeeding practices for smaller duration were not capable of responding with certainty about the precise interval of breastfeeding.

Participating women additionally reported whether they gave a preterm birth (<37th week), and their responses were additionally cross-checked by their medical files so as to have more accurate records regarding the precise week of gestation that preterm birth took place. Nevertheless, we detected that several data were lost regarding the precise week of preterm birth in the medical records, and several of them did not come to an agreement with the women’s responses, and thus, preterm birth was categorized as binary outcome, with childbirth either earlier than or after the 37th week of gestation. Measured childbirth weight data were collected by their mother’s gynecologists’ or hospitals’ medical files. Childbirth weight of was categorized as low (<2500 g), normal (2500–4000 g), and high (>4000 g).

Maternal smoking habits, educational level, economic status, age, and parity status were recovered by the given questionnaires according to mothers’ memory recall. Specifically, educational level was estimated based on the total years of education, and financial level was categorized based on the yearly family income as: 0, <5000; 1, 5000–10,000; 2, 10,000–15,000; 3, 15,000–20,000; 4, 20,000–25,000; and 5, >25,000 in euros. Financial level was additionally categorized as low for family annual income ≤ 10,000, medium for annual income ˃10,000 and ≤20,000, and high for annual income ˃ 20,000, all in euros.

The explaining guidelines were provided to the participating mothers by qualified dietitians and nutritionists concerning the accomplishment of questionnaires, and a thorough demonstration of the questions to support precise responses was made.

### 2.3. Statistical Analysis

Statistical analysis was accomplished by Student’s *t*-test for continuous variables followed normal distribution based on Kolmogorov–Smirnov test. All continuous variables followed normal distributions. Chi-square test was applied for categorical variables. The normally distributed quantitative variables are given as mean value ± standard deviation (SD), and the qualitative variables as absolute or relative frequencies. Multivariate logistic regression analysis was applied for assessing whether maternal GDM status is independently associated with sociodemographic and anthropometric characteristics and perinatal outcomes by adjusting for potential confounding factors. Differences were classified as significant at *p* < 0.05 and 95% confidence interval. The statistical analysis of the study data was accomplished by Statistica 10.0 software, Europe (Informer Technologies, Inc., Hamburg, Germany).

## 3. Results

### 3.1. Sociodemographic and Anthropometric Parameters and Perinatal Outcomes of the Study Population

The current survey included 5348 pre-school children at the age of 2–5 years old and their matched mothers, enrolled 2–5 years after delivery. Table 1 presents the descriptive statistics of the survey population. The mean age of the mothers was 33.7 ± 4.7 years (range: 22–46 years) at the time of pregnancy, and the mean age of their children was 4.06 ± 1.06 years (range: 2.0–5.5 years) at the time of study. Concerning children’s gender, 49.3% were males, and the remaining 50.7% were females. Regarding children’s BMI at age 2–5 years old, 16.4% of them were classified as overweight, 8.2% of them were classified as obese, and in total, 24.6% of the children were affected by overweight or obesity at the age of 2–5 years old. The BMI of the participating women prior to gestation was 22.7 ± 3.7 Kg/m^2^ (range: 15.9–37.6 Kg/m^2^). In fact, 17.5% of the women were affected by overweight, and 4.9% were affected by obesity prior to gestation based on BMI classification, and overall, an incidence of 22.4% regarding maternal overweight/obesity prior to gestation was recorded.

The mean years of maternal education were 14.6 ± 2.8 years (range: 0–17 years). Concerning economic status, 45.8% of mothers had low family annual income, 45.6% had medium family annual income, and 8.6% of them had high family annual income. In total, 25.5% of the mothers were smokers before pregnancy, and the remaining 74.5% of the mothers were never smokers. Further, 59.7% of the participants had no child since this was the first childbirth, and 40.3% of mothers had more than one child except for the childbirth included in this study. Moreover, 5.5% of the mothers were diagnosed with GDM, and 4.1% of the mothers were diagnosed with gestational hypertension. The mean maternal GWG was 13.8 ± 6.1 (range: 0–45 Kg). Preterm birth (<37th week) was noted in 30.1% of the participating mothers.

Half of participating women (49.8%) followed exclusive breastfeeding for a minimum period of 4 months (mean duration: 4.5 ± 1.9 months), and 50.2% of them did not follow exclusive breastfeeding for a minimum period of 4 months or did not breastfeed at all. Classifying children according to their birth weight, 8.2% of them were categorized as low newborn weight (< 500 g), 85.3% of them had normal newborn weight (2500–4000 g), and 6.5% of them had high newborn weight (>4000 g).

### 3.2. Maternal Gestational Diabetes Mellitus in Relation to Sociodemographic and Anthropometric Parameters and Perinatal Outcomes of the Study Population

Older women were at higher risk of developing GDM than younger women (Table 2, *p* < 0.0001). A significantly higher prevalence of delivering a female child was observed in women who developed GDM (Table 2, *p* = 0.0012). Childhood overweight and obesity at the age of 2–5 years old was considerably more often observed in the offspring of mothers who developed GDM than in the children of mothers without history of GDM (Table 2, *p* < 0.0001). Mothers who developed GDM were also more frequently affected by overweight or obesity pre-pregnancy compared to those that were not diagnosed with GDM (Table 2, *p* = 0.0155).

Low family annual income was considerably related with a higher incidence of maternal GDM (Table 2, *p* = 0.0065). Mothers who smoked were also more likely to be diagnosed with GDM than those who never smoked (Table 2, *p* = 0.0049). Multiparity was borderline-correlated with a greater GDM incidence (Table 2, *p* = 0.0552). Women who developed GDM showed a marginally lower prevalence of exclusive breastfeeding than those who were not diagnosed with GDM (Table 2, *p* = 0.0656). Maternal educational level and pregnancy-induced hypertension as well as childbirth weight were not associated GDM (Table 2, *p* ˃ 0.05).

### 3.3. Multivariate Regression Analysis for Gestational Diabetes Mellitus Status

In the multivariate logistic regression analysis, mothers developed GDM exhibited a 2-fold greater prevalence of delivering children that had overweight/obese BMI at the age of 2–5 years, independently of multiple confounders (Table 3, *p* = 0.0006). Mothers diagnosed with GDM showed a 27% greater incidence of delivering female children than male ones (Table 3, *p* = 0.0050).

Older women also showed a 29% greater risk of being diagnosed with GDM than younger ones (Table 3, *p* = 0.0027). Mothers affected by overweight or obesity before pregnancy exhibited more than a 30% greater likelihood of developing GDM (Table 3, *p* = 0.0326). Smokers also showed a 54% higher probability of developing GDM than non-smokers (Table 3, *p* = 0.0204). Mothers diagnosed with GDM showed a 77% greater prevalence of preterm birth compared to those without a history of GDM (Table 3, *p* = 0.0135). Family economic status, parity, educational level, gestational weight gain, breastfeeding practices, and childbirth weight were not considerably related to the probability of developing GDM in multivariate analysis (Table 3, *p* ˃ 0.05).

## 4. Discussion

In the current survey, 24.6% of the children aged 2–5 years old were either affected by overweight or obesity. Childhood obesity levels in Greece are high, and according to the COSI study data, Greece leads the way in Europe for severe obesity in children [34]. In pre-school aged children, the rate of overweight and obesity is 21–22% [8,35].

Children of mothers that developed GDM exhibited greater probability of overweight or obesity, with the relationship between GDM and offspring’s weight status being independent of confounding factors. In Europe, the incidence of GDM has been estimated to be 5.4% (range: 3.8–7.8%), which is in line with our results [36]. Moreover, the recently published study by Gao et al. [37] found that even maternal insulin resistance, assessed via elevated HOMA-IR, was related to an enhanced likelihood for the children to be overweight during the first two years of life [37]. The HAPO survey [38,39] findings, with data from 10 countries and 4832 children, are also in line with our findings. In utero glucose levels lead to higher adiposity in childhood and increased waist circumference. Furthermore, a novel survey by Choi et al. [40] is in line with our findings, as the authors observed that maternal obesity before gestation and GDM further raise the probability of childhood obesity at 5 years [40]. Another recent retrospective study by Ardic et al. [41] also found that GDM increases the probability for childhood obesity at the ages of 2 and 3 years old.

The proposed mechanisms behind this association are the exposure to higher glucose levels in utero [38,39], reduced insulin sensitivity due to maternal hyperleptinemia [42], epigenetic programming [43], as well as the altered maternal gut microbiome due to GDM [44]. It should be noted that maternal BMI before gestation is another important factor that incorporates both genetic predisposition and shared obesogenic environment [45].

It is of great concern that childhood obesity increases the probability of obesity through the different stages of life [46]. More to the point, 55% of children affected by obesity become adolescents with obesity, and 80% of these adolescents will still be affected by obesity in adulthood [47]. Additionally, childhood obesity can increase the risk of cardiometabolic diseases and cancer in adulthood [48,49].

In our analysis, mothers affected by overweight or obesity before gestation were at 30% higher risk of developing GDM than those with normal weight. Indeed, it is very well established in the literature that higher BMI status raises the probability of glucose intolerance and GDM [15,16,17]. In fact, a dose–response association between BMI and the probability of GDM development was discerned, with the risk increasing by 4% for every unit of increase on BMI [16], while the probability of GDM in women affected by overweight or obesity tends to 23% [17]. Accordingly, in a meta-analysis of 39 cohorts from Europe, North America, and Oceania, women affected by obesity with high GWG exhibited the greatest likelihood of gestational complications such as pregnancy-induced hypertensive disorders, GDM, and large for gestational age at childbirth [50]. In accordance with the above, we demonstrated that mothers diagnosed with GDM exhibited greater GWG; nevertheless, this relationship was considerably attenuated after adjusting for potential confounding factors.

Several studies also suggested that GDM may increase the likelihood of increased childbirth weight and large for gestational age and macrosomia [18]. Recently, a prospective, hospital-based cohort study showed that GDM increased the odds of preterm birth, higher birthweight, and large for gestational age [51]. Although we recorded an elevated probability of preterm birth in mothers diagnosed with GDM, there was no relationship between GDM and childbirth weight, which may be ascribed to the rather modest prevalence of GDM in our survey population.

Interestingly, in our study, women who developed GDM exhibited a 27% greater incidence of delivering female children than male ones. Recent findings have demonstrated that fetal sex may exert a role on maternal insulin sensitivity [52]. In GDM, the placenta is affected, and different gene expression takes place, with fetal sex playing an important role [53,54]. Moreover, a lipotoxic placenta environment has been established in obese mothers, with enhanced inflammatory and oxidation stress. The above conditions may change mitochondrial function, producing reactive oxygen species, which may result in placenta dysfunction and diminished gestation outcomes [55]. In addition, placenta metabolome analysis of pregnant women affected by obesity revealed differences in metabolites implicated in antioxidant defense systems, nucleotide production, as well as lipid biosynthesis and energy production, supporting a shift to greater placenta metabolism [56]. These metabolic signatures in the placentas of women affected by obesity could indicate alterations occurring in the intrauterine metabolic environment, which could affect the risk of several diseases during adulthood [56].

Regarding GDM and fetal sex, Hooks et al. [57] found that in women with GDM, having a male fetus raises the probability of preterm birth [55], while Seghieri et al. [58] found that having a male fetus raises the likelihood of GDM, and in pregnancies with a female fetus, maternal obesity before gestation increases the probability of GDM. Another recent study found that having a male fetus is associated with higher insulin sensitivity [52]. Moreover, Mando et al. explored the potential effect of GWG on fetal/placental ratio in overweight with different fetal gender and found different placenta adaptation depending on fetal sex, with substantial alterations merely in female fetuses [59]. The above could be part of a female-specific approach intending to guarantee survival if another adverse outcome appears [59].

Regarding preterm birth, mothers diagnosed with GDM had a 77% higher probability of preterm birth compared to mothers who did not develop GDM. Several studies have shown that GDM increases the risk for preterm birth by 30–42% [60,61]. The difference in the risk might be ascribed to the fact that our survey sample is quite smaller that the above studies.

Women’s age is considered a risk factor for developing GDM [62], and our study confirms that older mothers had a 29% greater likelihood of developing GDM compared to younger ones. Smokers also showed a 54% higher probability of developing GDM than non-smokers. and although earlier meta-analyses showed no association between smoking and GDM [63], more recent studies have revealed a relationship with smoking [64,65] and even passive smoking [66].

Breastfeeding also exerts beneficial effects on mothers’ post-natal weight loss [67], being related with decreased probability of childhood obesity by 22% [68,69] in a dose-response manner [70]. In addition, in spite of the advantages of breastfeeding for both children’s and mothers’ health and body weight, women affected by obesity are less prone to apply breastfeeding practices to their children [71,72]. Moreover, the incidence of breastfeeding among GDM mothers is far less than optimal. Based on the WHO, only over one-third (34.8%) of GDM mothers apply breastfeeding practices for their children, and the proportion is even lower in developing countries [73]. However, we found only a marginal association between breastfeeding and GDM incidence, which was further attenuated after adjustment for confounders that may be ascribed to the rather low prevalence of GDM in our survey population.

It should be mentioned that there are some limitations in our survey. BMI is considered a crucial indicator to classify mothers as overweight or obese. However, direct techniques evaluating body fat quantity and distribution are needed to expand and confirm the present findings. Moreover, memory bias was essential in our survey because some probable risk factors were self-reported by the participating women. Hence, no conclusive evidence concerning causality can be derived because of the cross-sectional design of the present study in spite of its nationally representative nature. Another limitation of our study deals with the fact that it did recorded neither feeding practices of mothers during their pregnancy nor eating habits of their children beyond the breastfeeding period. In this aspect, specific nutritional approaches such as the Mediterranean diet have been related to a significant decrease of certain gestational complications, such as GDM, overweight or obesity, sleep quality, childbirth difficulties, urinary tract infections, and alterations in fetal growth as well as perinatal problems including childbirth weight, prematurity, gastroschisis, and other childhood complications [74]. There is also substantial evidence that healthy dietary patterns such as the Mediterranean diet can decrease the risk of developing overweight and obesity through childhood up to adolescence [75].

Furthermore, despite a thorough effort adjust confounding factors, we acknowledge the likelihood of immeasurable confounding factors. Nevertheless, our survey advantage is the comparatively high and representative survey population given that it comprised women and their paired children from nine geographically different regions of Greece, including urban, rural, and island areas. The survey sample was adequately high and consisted of a Caucasian population, and thus, its representativeness may be judged as quite sufficient. Hence, the present findings may well be extrapolated away from the Greek population to other Caucasian peoples of other ethnicities. Nevertheless, further studies need to be conducted on other ethnic groups that could exhibit various differences regarding genetic background, sociodemographic, and lifestyle factors.

## 5. Conclusions

GDM is a serious public health issue worldwide, with prolonged complications for both the mother and its child. The present study supported evidence at a Caucasian population-based level that GDM raises the risk for childhood overweight/obesity in the early years of the offspring’s life, which further may raise the risk for adolescent and adulthood obesity. The strong relationship between mothers’ GDM and childhood overweight/obesity is independent of multiple confounding factors. The risk of GDM is greater in women with higher weight status and those who smoke; hence, public health approaches and policies need to promote the important role of pre-pregnancy weight, GWG, and smoking status as well as the importance of a good glycemic control throughout pregnancy. Future studies are recommended to explore whether healthy dietary patterns during pregnancy may reduce GDM risk, also highlighting the need to improve eating habits at the early years of children’s life to decrease the risk of overweight and obesity and the related complications at the next stages of life.

## Figures and Tables

**Table 1:** Descriptive statistics of the study population.

Characteristics (n = 5348)	Descriptive Statistics
Maternal age (mean ± SD; years) at the time of pregnancy	33.7 ± 4.7
Children age (mean ± SD; years) at the time of study	4.06 ± 1.06
Children gender (n, %)	
Male	2639 (49.3%)
Female	2709 (50.7%)
Children BMI status at 2–5 years old (n, %)	
Normal weight	4033 (75.4%)
Overweight	878 (16.4%)
Obese	437 (8.2%)
Maternal pre-pregnancy BMI status (n, %)	
Underweight and normal weight	4148 (77.6%)
Overweight	936 (17.5%)
Obese	264 (4.9%)
Maternal education level (mean ± SD; years)	14.6 ± 2.8
Family economic status (n, %)	
Low	2449 (45.8%)
Medium	2440 (45.6%)
High	459 (8.6%)
Maternal smoking habits (n, %)	
No smokers	3984 (74.5%)
Smokers	1364 (25.5%)
Parity (n, %)	
Nulliparity	3193 (59.7%)
Multiparity	2155 (40.3%)
Maternal gestational weigh gain (mean ± SD; Kg)	13.8 ± 6.1
Preterm birth (<37th week; n, %)	
No	3739 (69.9%)
Yes	1609 (30.1%)
Maternal gestational diabetes (n, %)	
No	5052 (94.5%)
Yes	235 (5.5%)
Maternal gestational hypertension (n, %)	
No	5129 (95.9%)
Yes	219 (4.1%)
Exclusive breastfeeding (n, %)	
No	2689 (50.2%)
Yes	2659 (49.8%)
Childbirth weight (n, %)	
Low newborn weight (<2500 g)	436 (8.2%)
Normal newborn weight (2500–4000 g)	4564 (85.3%)
High newborn weight (>4000 g)	348 (6.5%)

**Table 2:** Associations of maternal gestational diabetes status with sociodemographic and anthropometric parameters and perinatal outcomes of the study population.

Characteristics (n = 5348)	Gestational Diabetes Mellitus		
	No (94.5%)	Yes (5.5%)	*p*-Value
Maternal age (mean ± SD; years) at the time of pregnancy	33.4 ± 4.7	36.1 ± 4.8	*p* < 0.0001
Children gender (n, %)			*p* = 0.0012
Male	2520 (49.9)	119 (40.2)	
Female	2532 (50.1)	177 (59.8)	
Children BMI status (n, %)			*p* < 0.0001
Normal weight	3890 (77.0)	143 (48.3)	
Overweight	795 (15.7)	83 (28.0)	
Obese	367 (7.3)	70 (23.7)	
Maternal pre-pregnancy BMI status (n, %)			*p* = 0.0155
Underweight and normal weight	3934 (77.9)	214 (72.3)	
Overweight and obese	1118 (22.1)	82 (27.7)	
Maternal education level (mean ± SD; years)	14.6 ± 2.8	14.7 ± 2.8	*p* = 0.6765
Family economic status (n, %)			*p* = 0.0065
Low	2300 (45.5)	149 (50.3)	
Medium	2304 (45.6)	136 (46.0)	
High	448 (8.9)	11 (3.7)	
Maternal smoking habits (n, %)			*p* = 0.0049
No smokers	3784 (74.9)	200 (67.6)	
Smokers	1268 (25.1)	96 (32.4)	
Parity (n, %)			*p* = 0.0552
Nulliparity	3032 (60.0)	161 (54.4)	
Multiparity	2020 (40.0)	135 (45.6)	
Maternal gestational weigh gain (mean ± SD; Kg)	13.0 ± 5.0	13.8 ± 6.2	*p* = 0.0323
Preterm birth (<37th week, n, %)			*p* = 0.0002
No	3560 (70.5)	179 (60.5)	
Yes	1492 (29.5)	117 (39.5)	
Maternal gestational hypertension (n, %)			*p* = 0.1647
No	4839 (95.8)	290 (98.0)	
Yes	213 (4.2)	6 (2.0)	
Exclusive breastfeeding (n, %)			*p* = 0.0656
No	2527 (50.0)	162 (54.8)	
Yes	2525 (50.0)	134 (45.3)	
Childbirth weight (n, %)			*p* = 0.3115
Low newborn weight (<2500 g)	405 (8.0)	31 (10.5)	
Normal newborn weight (2500–4000 g)	4321 (85.5)	243 (82.1)	
High newborn weight (>4000 g)	326 (7.5)	22 (7.4)	

**Table 3:** Multivariate logistic regression analysis for evaluating whether maternal gestational diabetes mellitus was independently related to sociodemographic and anthropometric characteristics and perinatal outcomes after adjusting for multiple confounding factors.

Characteristics	Gestational Diabetes Mellitus	
	OR * (95% CI **)	*p*-Value
Children BMI status (Normal weight/overweight or obese)	2.13 (1.94–2.31)	*p* = 0.0006
Children gender (Male/female)	1.27 (0.88–1.69)	*p* = 0.0050
Maternal age (Below/over mean value) at the time of pregnancy	1.29 (0.92–1.64)	*p* = 0.0027
Maternal pre-pregnancy BMI status (Underweight and normal weigh/overweight and obese)	1.30 (0.79–1.92)	*p* = 0.0326
Maternal education level (Below/over mean value)	0.97 (0.22–1.87)	*p* = 0.8502
Family economic status (Low or medium/high)	0.89 (0.24–1.71)	*p* = 0.2147
Smoking habits (No/yes)	1.54 (1.16–1.97)	*p* = 0.0204
Parity (Nulliparity/multiparity)	1.26 (0.69–1.80)	*p* = 0.2813
Maternal gestational weigh gain (Below/over mean value)	1.40 (0.61–2.12)	*p* = 0.3172
Preterm birth (No/yes)	1.77 (1.41–2.16)	*p* = 0.0135
Maternal gestational hypertension (No/yes)	1.14 (0.43–1.96)	*p* = 0.3804
Exclusive breastfeeding (No/yes)	1.31 (0.86–1.89)	*p* = 0.0932
Childbirth weight (Low or normal/high)	1.19 (0.52–1.91))	*p* = 0.4311

* OR, odds ratio; ** CI, confidence interval.

## Data Availability

Data available upon request from corresponding authors.
